# Therapeutic Potential of Natural Plants Against Non-Alcoholic Fatty Liver Disease: Targeting the Interplay Between Gut Microbiota and Bile Acids

**DOI:** 10.3389/fcimb.2022.854879

**Published:** 2022-03-09

**Authors:** QinMei Sun, Xin Xin, ZiMing An, YiYang Hu, Qin Feng

**Affiliations:** ^1^ Institute of Liver Diseases, Shuguang Hospital Affiliated to Shanghai University of Traditional Chinese Medicine, Shanghai, China; ^2^ Shanghai Key Laboratory of Traditional Chinese Clinical Medicine, Shanghai, China; ^3^ Key Laboratory of Liver and Kidney Diseases, Shanghai University of Traditional Chinese Medicine, Ministry of Education, Shanghai, China

**Keywords:** natural plants, active ingredients, non-alcoholic fatty liver disease, gut microbiota, bile acids

## Abstract

Non-alcoholic fatty liver disease (NAFLD) remains a common disease with a significant health and economic burden worldwide. The gut microbiota (GM) and bile acids (BAs), which play important roles in the gut-liver axis, have been confirmed to jointly participate in the development of NAFLD. GM not only regulate bile acids’ synthesis, transport, and reabsorption by regulating other metabolites (such as trimetlyl amine oxide, butyrate), but also regulate dehydrogenation, dehydroxylation and desulfurization of bile acids. Meanwhile, disordered bile acids influence the gut microbiota mainly through promoting the bacterial death and lowering the microbial diversity. Although weight loss and lifestyle changes are effective in the treatment of NAFLD, the acceptability and compliance of patients are poor. Recently, increasing natural plants and their active ingredients have been proved to alleviate NAFLD by modulating the joint action of gut microbiota and bile acids, and considered to be promising potential candidates. In this review, we discuss the efficacy of natural plants in treating NAFLD in the context of their regulation of the complex interplay between the gut microbiota and bile acids, the crosstalk of which has been shown to significantly promote the progression of NAFLD. Herein, we summarize the prior work on this topic and further suggest future research directions in the field.

## 1 Introduction

Non-alcoholic fatty liver disease (NAFLD), characterized by excessive lipid deposition in hepatocytes, has become the most common chronic liver disease worldwide. Clinically, the spectrum of histopathology ranges from simple steatosis (NAFL) and non-alcoholic steatohepatitis (NASH) to liver cirrhosis (Friedman et al., 2018b; Powell et al., 2021). Currently, the global incidence of NAFLD has greatly increased to an estimated prevalence of 25% among adults (NCD Risk Factor Collaboration, 2017; Hales et al., 2018; Zhou et al., 2019a). Until now, the best characterization of the mechanisms underlying NAFLD is provided in the “two hit” theory proposed by Day in 1998; this theory suggests that on the basis of hepatic lipid accumulation caused by developed insulin resistance, the “two hit” from other factors is required to trigger the pathological procession of NAFLD (Day and James, 1998; Friedman et al., 2018b). However, the exact mechanism underlying metabolic disorders in NAFLD remains unclear. The “gut-liver axis,” first proposed by Marshall in 1998, is essential in the regulation of systemic metabolism, gut hormone release, and the immune response (Marshall, 1998). The gut microbiota (GM) and bile acids (BAs), which play important roles in the gut-liver axis, have been confirmed to jointly participate in the development of NAFLD (Chavez-Talavera et al., 2017; Kolodziejczyk et al., 2019; Aron-Wisnewsky et al., 2020).

The GM is defined as a complex and dynamic microbial ecosystem in the gut, composed of symbiotic bacteria, archaea, fungi, and viruses, (Zmora et al., 2019). Several studies have shown that the GM and its metabolites could be used as key signaling factors to regulate the host’s glucose and lipid metabolism, insulin resistance, immunity, and inflammation in NAFLD (Aron-Wisnewsky et al., 2020). BAs, a metabolite of GM, are a class of amphiphilic molecules synthesized from cholesterol (Li and Chiang, 2014). Significant evidence has suggested that BAs could participate in NAFLD by regulating glucose and lipid metabolism and energy homeostasis (Arab et al., 2017). Meanwhile, the crosstalk between the GM and BAs can significantly promote the progression of NAFLD (Jia et al., 2018; Winston and Theriot, 2020; Agus et al., 2021). Thus, the interplay between GM and BAs may be a promising target for the prevention and treatment of NAFLD.

Natural plants, including plants and active ingredients, have been proven as effective treatments of NAFLD (Liu et al., 2017; Leng et al., 2020; Xin et al., 2021). The therapeutic effects of natural plants have attracted increasing attention. Furthermore, the potential regulatory effects of natural plants on intestinal dysbacteriosis and BA metabolism have been reported (Meng et al., 2018; Leng et al., 2020). Thus, this review aimed to discuss the efficacy of natural plants, which act by regulating the interplay between GM and BAs, against NAFLD.

## 2 The Interplay of GM-BAs in NAFLD

The human GM is rich in various species, including those of *Firmicutes, Bacteroides, Proteobacteria*, and *Actinomycetes* in the gut (Eckburg et al., 2005). Early evidence linking gut dysbiosis and NAFLD has shown that the abundance of gram-negative bacteria increases, whereas that of gram-positive bacteria decreases during the course of NAFLD, indicating that microbial populations are altered in NAFLD patients (Ley et al., 2006). A large number of metabolites, including BAs, lipopolysaccharides, short-chain fatty acids (SCFAs), and inflammatory factors, are produced following host–microorganism interactions. The content of BAs, especially secondary BAs, increases significantly in the serum of hosts with NAFLD (Jiao et al., 2018). Meanwhile, the interplay between GM and BAs has been shown to maintain host homeostasis in NAFLD (Jiao et al., 2021). When colonizing germ-free mice with the feces of wild type or humans, the total BA content was obviously reduced (Sayin et al., 2013).

### 2.1 Microbial Regulation of BAs in NAFLD

**Figure 1:**
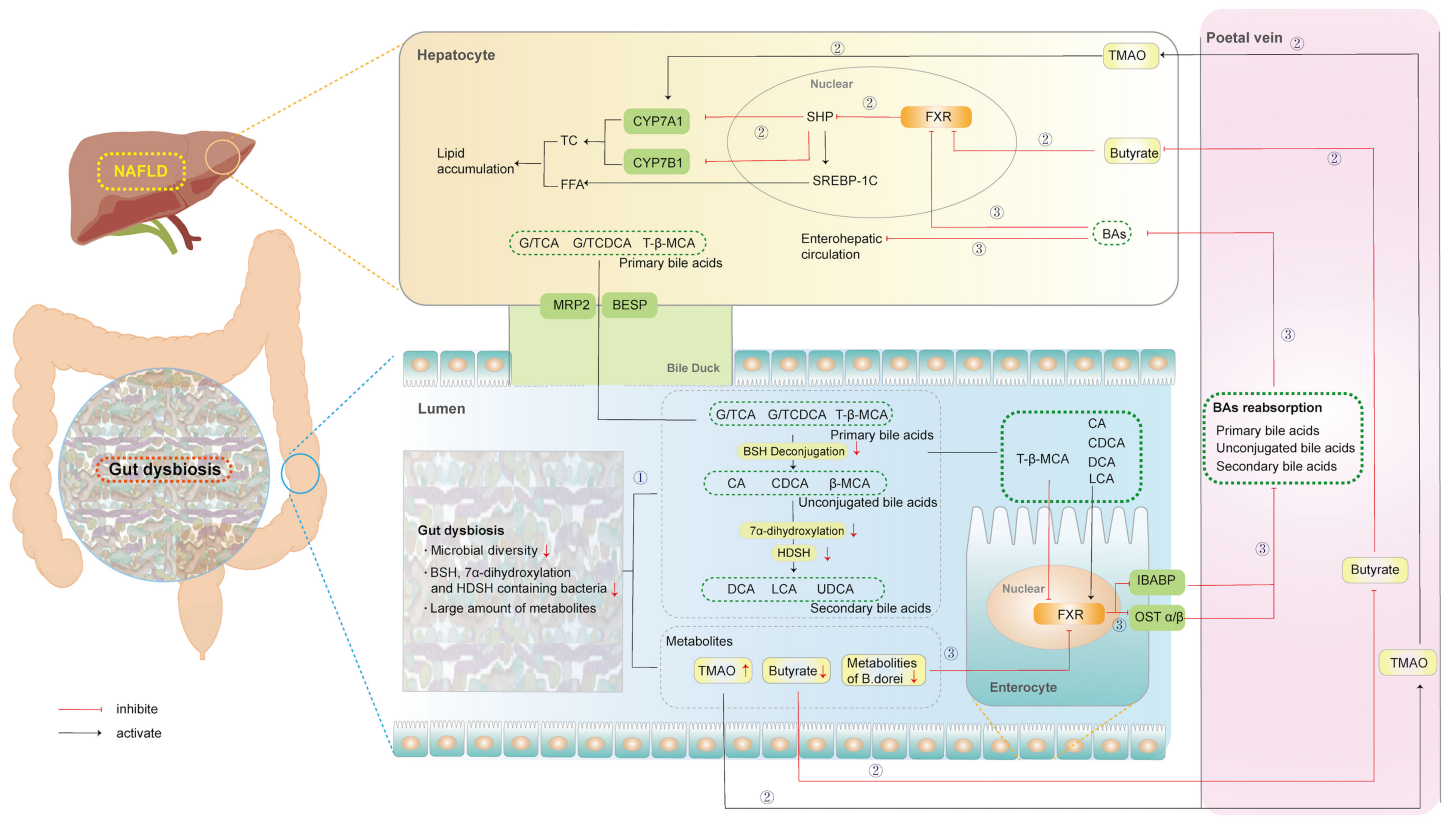
Microbial regulation of Bile Acids in NAFLD. ①: Microbial modification of primary BAs to secondary BAs. ②: Microbial regulation of BAs synthesis. ③: Microbial regulation of BAs transport, and reabsorption. (BESP, bile salt export pump; MRP2, multidrug resistance protien 2; BAs, bile acids; BSH, bile salt hydrolase; β-MCA, β-murocholic acid; CA, Cholic acid; CDCA, Chenodeoxycholic acid; CYP7A1, cytochrome P450 family 27 subfamily A member 1; CYP7B1, cytochrome P450 family 7 subfamily B member 1; DCA, deoxycholic acid; FXR, farnesoid X receptor; FFA, free fatty acid; IBABP, ileal bile acid binding protein; LCA, lithocholic acid; NAFLD, non-alcoholic fatty liver disease; SHP, small heterodimer partner; SREBP-1, sterol regulatory element binding protein-1; TβMCA, tauro-β-muricholic acid; TC, total cholesterol; TMAO, trimethylamine oxide; UDCA, ursodeoxycholic).

The GM regulates the synthesis, transport, and reabsorption of BAs *via* regulation of metabolites and the farnesoid X receptor (FXR). Moreover, the GM can also modify primary BAs to secondary BAs with enzymes such as bile salt hydrolase (BSH) through dehydrogenation, dehydroxylation, and desulfurization. All of this research has been clarified and discussed below, with the mechanisms represented in **Figure 1**.

#### 2.1.1 Microbial Regulation of BAs Synthesis, Transport, and Reabsorption

The synthesis of BAs is complex, including reaction steps catalyzed by more than a dozen enzymes. BAs are synthesized in pericentral hepatocytes and can be fulfilled *via* two different pathways. Specifically, the classical pathway, which accounts for 75% of BA synthesis, is initiated by the 7a-hydroxylation of cholesterol catalyzed by cytochrome P450 family 27 subfamily A member 1 (CYP7A1). In a case-control study, trimethylamine oxide (TMAO), the metabolites from GM, significantly increased BA synthesis by upregulating hepatic *CYP7A1* mRNA levels in patients with NAFLD (Tan et al., 2019). Correspondingly, the alternative pathway is initiated by the 27-hydroxycholesterol of cholesterol catalyzed by CYP27A1 and further hydroxylated by cytochrome P450 family 7 subfamily B member 1 (CYP7B1) in the liver. This pathway accounts for approximately 9–25% of BA synthesis. After antibiotic treatment, hepatic CYP7B1 was upregulated in high-fat diet (HFD)-fed hamsters, contributing to a more hydrophilic BA profile with increased tauro-β-muricholic acid (TβMCA) (Sun et al., 2019a). Therefore, the GM regulates the expression of several enzymes in the BA synthesis pathway to promote NAFLD. Furthermore, the alternative pathway predominantly generates Chenodeoxycholic acid (CDCA), whereas the classical pathway generates both CDCA and Cholic acid (CA), which are called primary BAs (Chiang, 2013). Rodents also produce α-murocholic acid (α-MCA) and β-murocholic acid (β-MCA) as primary BAs. Sheng confirmed that butyrate, the metabolite of GM, significantly decreased the content of β-MCA by activating hepatic FXR-small heterodimer partner (SHP) signaling in HFD-fed mice (Sheng et al., 2017). However, the regulation of β-MCA by GM may be attributed to unknown enzymes.

Independent of the synthetic route, the carboxylic acid group from CA/CDCA conjugated the peroxisomes of glycine or taurine to form conjugated BAs (G/TCA and G/TCDCA, respectively) in humans (Jia et al., 2018). This conjugation with taurine alone occurred to form TCA/TCDCA in rodents. Subsequently, conjugated BAs are actively transported into bile *via* the bile salt export pump (BSEP) and may be stored in the gall-bladder until being released into the duodenum after ingestion of a meal. Approximately 95% of BAs are reabsorbed from the intestine, predominantly as conjugated BAs in the distal ileum, by the apical sodium-dependent bile acid transporter (ASBT), and recirculate *via* the portal vein to the liver, from where they are secreted again (Ticho et al., 2019). Treatment with FXR-stimulatory *Bacteroides dorei*-derived metabolites could strongly induce ileal bile acid binding protein (IBABP) and organic solute transporter (OST) α, thereby improving the obesity phenotype, including body weight gain, liver damage, and lipid metabolism in HFD-fed mice (Zhang et al., 2015). Overall, GM can regulate the synthesis, transport, and reabsorption of BAs *via* its metabolites or FXR in the progression of NAFLD.

#### 2.1.2 Microbial Modification of Primary BAs to Secondary BAs

In addition, GM participates in the biotransformation of BAs *via* the catalysis of microbial enzymes, thereby changing the composition of BA pools in NAFLD. Hydrolysis mediated by BSH transforms conjugated BAs into unconjugated BAs. BSH, an intracellular enzyme encoded by the *bsh* gene (Song et al., 2019), has been extensively identified in gastrointestinal microorganisms, including *Lactobacillus*, *Bifidobacterium*, *Enterococcus*, *Clostridium*, and *Bacteroides* (Ridlon et al., 2006). Different intestinal bacteria are associated with different BSH activities. The levels of taurine and glycine-metabolizing bacteria that express BSH enzymes were enhanced in the gut of patients with NAFLD, significantly increasing the production of secondary BAs (Jiao et al., 2018).

Next, unconjugated BAs were further transformed into deoxycholic acid (DCA) and lithocholic acid (LCA) *via* 7α-dehydroxylation or ursodeoxycholic (UDCA) by 7-alpha-hydroxysteroid dehydrogenase (HSDH). Sydro et al. indicated a clear association between the 7α-dehydrogenase related to *Bacteroides* and *Lactobacillus*, and the primary conjugated BA composition in patients with NASH (Sydor et al., 2020). Recently, Yu et al. analyzed the associations between GM and the concentrations of primary and secondary BAs in fecal samples of children with NAFLD. Further, a reduction in *Eubacterium* and *Ruminococcaceae* bacteria, which express BSH and 7α-dehydroxylation, was significantly positively correlated with the level of fecal LCA (Yu et al., 2021).

Furthermore, the main conclusions of the previous studies are consistent with those observed in rodents. In HFD-fed mice, higher abundance levels of the genus *Lactobacillus* and increased BSH activity resulted in decreased levels of TβMCA, a substrate of BSH, and a potent FXR antagonist (Gonzalez et al., 2016). Another study also confirmed that an 8 week HFD significantly increased abundance levels of *Extibactermuris*, related to 7α-dehydroxylation, metabolizing CA to DCA in mice (Streidl et al., 2021). Similarly, *Bacteroides* expressing HSDH and 7-alpha HSDH were enriched to further modify unconjugated BAs into UDCA in the gut microbiome of HFD-fed rats (Jiao et al., 2018).

### 2.2 BAs Influence the GM in NAFLD

**Figure 2:**
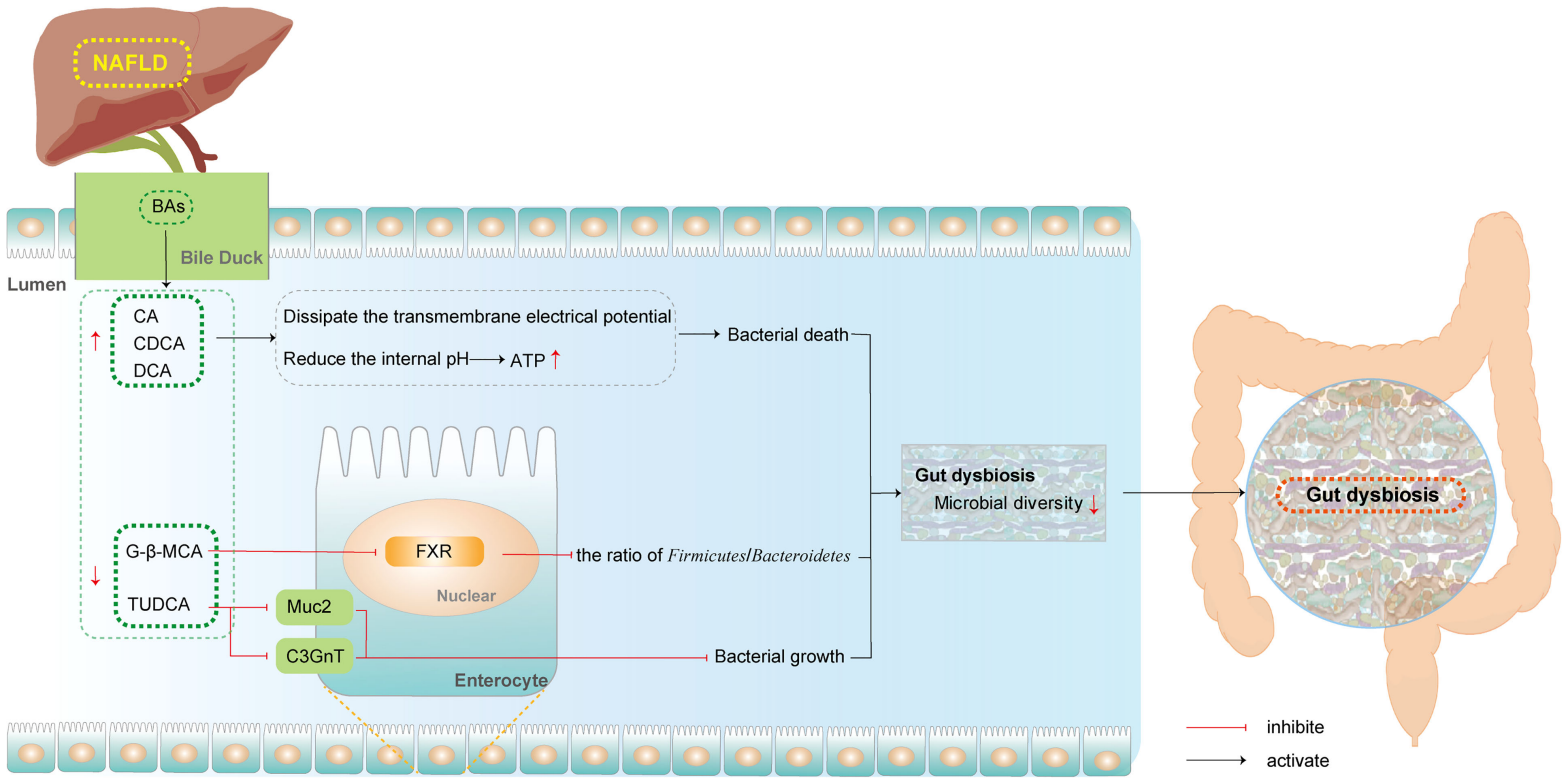
BAs influence GM in NAFLD. (BAs, bile acids; CA, Cholic acid; CDCA, Chenodeoxycholic acid; C3GnT, Core 3 O-glycan; DCA, deoxycholic acid; FXR, farnesoid X receptor; Muc2, mucin 2; NAFLD, non-alcoholic fatty liver disease; G-β-MCA, glycine-β-muricholic acid; TUDCA, tauro-ursodeoxycholic).

Despite this, the interplay between the GM and BAs is not a one-way street. BAs can further influence the structure and function of the GM. After administration of obeticholic acid (OCA), the levels of gram-positive bacteria increased in humans, whereas that of *Firmicutes* bacteria increased in mice (Friedman et al., 2018a). Sevelamer, a BA sequestrant, reversed this increase in *Lactobacillus* and decreased the levels of *Desulfovibrio* in HFD-fed mice (Takahashi et al., 2020). Other promising studies have shown that BAs exert direct and indirect effects on the GM (**Figure 2**).

#### 2.2.1 BAs Directly Affect the GM

The direct action of BAs on GM has been shown to promote cell death *via* membrane regulation. In the intestinal lumen, BAs exert direct antimicrobial activity based on their detergent properties to shape the GM (Schubert et al., 2017). Free bile acids (FBAs), including CA, DCA, and CDCA, dissipated the transmembrane electrical potential (DeltaPsi). In the human intestine, the populations of *Lactobacillus* and *Bifidobacterium* are controlled in part by the accumulation of CA. As this accumulation must occur during bacterial growth in the intestine, growth inhibition may be associated with the accumulation of FBAs. Taking these observations together, although FBAs disturb the membrane integrity, leading to leakage of proton and other cellular components, they also reduced the internal pH levels of bacteria with rapid and stepwise kinetics and, at certain concentrations, dissipated DeltapH. High concentrations of BAs can decrease the internal pH of *Lactobacilli* and *Bifidobacteria*, and reduce the production of ATP, ultimately inducing bacterial death (Kurdi et al., 2006).

#### 2.2.2 BAs Indirectly Affect the GM

In addition to exerting strong direct effect, the indirect effects by which BAs regulate GM through signaling factors also have significant consequences. Basic experiments have confirmed the role of FXR in regulating the structure of intestinal bacteria (Inagaki et al., 2006). Zhang et al. confirmed that oral G-β-MCA administration altered the gut microbial community structure, notably reducing the ratio of *Firmicutes* to *Bacteroidetes* in NASH mice. This revealed that metabolic improvement was dependent on intestinal FXR (Zhang et al., 2016). In addition, TUDCA has been shown to increase the *Bacteroidetes/Firmicutes* ratio and the abundance of *Faecalibacterium* and *Akkermansia* in mice with NAFLD. The growth of bacterial genera might be related to the upregulation of mucin 2 (Muc2) and Core 3 O-glycan (C3GnT), which provide a substrate for the growth of intestinal bacteria (Wang et al., 2018).

## 3 Natural Plants Regulate the Interplay Between GM and BAs in the Treatment of NAFLD

Although weight loss and lifestyle changes are effective in the treatment of NAFLD (Vilar-Gomez et al., 2015; Khoo et al., 2017), the acceptability and compliance of patients are poor. Currently, the use of synthetic drugs for the treatment of NAFLD remains limited. Despite the numerous options for the tested pharmaceuticals, none of them are satisfactory in treating NAFLD. However, increasing evidence has indicated that natural plants efficiently exhibit anti-NAFLD properties, with lower toxicity and side effects, and may be useful in regulating the joint action of GM and BAs (Liu et al., 2017). Therefore, we summarized the new studies on Natural plants that have indicated that they exert anti-NAFLD properties by modulating the interplay between GM and BAs. All these are clarified and discussed below, with the mechanisms presented in **Tables 1**, **2**.

**Table 1:** Natural plants regulate the interplay between GM and BAs in the treatment of NAFLD.

Plants		Dosage and administration	Models	Main Effects on NAFLD	Main Effects on Gut Microbiota	Main Effects on Bile Acids	Reference
Plant Foods	Pu-erh tea	450 mg/kg/day in drink for 4, 22, 42 weeks	HFD-diet C57BL/6J male mice	body weight ↓ hepatic TC ↓, TG ↓ serum TC ↓, TG ↓	1.*Lactobacillus, Bacillus, Streptococcus, Lactococcus* ↓	serum TCDCA, TUDCA, GCDCA, GUDCA↑ fecal TCDCA, TUDCA ↑	(Huang et al., 2019)
	Huangjinya black tea	150, 300 mg/kg/day by gavage for 9 weeks	HFD-diet C57BL/6J male mice	body weight ↓, liver weight ↓ hepatic TG ↓, FFA ↓, TC ↓ hepatic lipid droplet ↓	1.GM disorders reversed	total BAs ↓ LCA/CDCA ↓, DCA/CA ↑	(Xu et al., 2020)
	Oat-based food	4.33 ± 0.17 g/mouse/day oat and 1.66 ± 0.07 g/mouse/day tartary buckwheat in diet for 30 days	HCD-diet Hamster male mice	hepatic TC ↓, TG ↓ serum TC ↓, TG ↓, LDL-C ↓	Erysipelotrichaceae, Ruminococcaceae, Lachnospiraceae ↓ Eubacteriaceae ↑	1. Fecal total BAs ↑	(Sun et al., 2019b)
	Wheat gluten	10.8 ± 0.48 g/mouse/day in diet for 5 weeks	HCD-diet Hamster male mice	body weight ↓ hepatic TC ↓, TG ↓ serum TC ↓, TG ↓, LDL-C ↓, HDL-C ↑	Firmicutes, Erysipelotrichaceae↓ Bateroidetes, Bacteroidales_S24-7_group, Ruminococcaceae ↑	1. Fecal total BAs ↑	(Liang et al., 2019)
	Lentils	62.5 mg/rat/day in drink for 71 days	HCD-diet SD Rat male	1.serum TC ↓, LDL↓, HDL↑	1.*Bifidobacterium spp* ↑	1. Fecal total BAs ↑	(Micioni Di Bonaventura et al., 2017)
	Pea pods	0.9g/rat/day in diet for 4 weeks	HS-diet SD Rat	1.serum TC ↓, TG↓	1.*Bifidobacterial*↑	1. Fecal total BAs ↑	(Inagaki et al., 2016)
	Wild Melon Seed Oil	42 ± 1.8, 85.5 ± 1.8 mg/mouse/day in diet for 6 weeks	HCD-diet Hamsters male mice	body weight, liver weight↓ hepatic TC ↓ serum TC ↓, TG ↓	1.*Eubacteriaceae*, *Clostridiales_vadinBB*60_group, *Ruminococcaceae*, *Streptococcaceae*, *Desulfovibrionaceae* ↑	1. Fecal total BAs ↑	(Hao et al., 2020)
	Sacha inchi oil	0.5, 1, 1.5 ml/kg/day by gavage for 8 weeks	HFD-diet SD Rat	hepatic fat accumulation ↓ hepatic adipocyte size ↓ serum ALT ↓, AST ↓ serum TC ↓, TG ↓, LDL-C↓	Enterobacteriaceae, Escherichia ↓ Roseburia, Turicibacter, Butyrivibrio ↑	1. CA, GCA, TCDCA, TCA ↓	(Li et al., 2020)
	Grape	5g/kg/day in diet for 13 weeks	HFFD-diet C57BL/6J male mice	body weight ↓ hepatic lipid droplet ↓ hepatic inflammation ↓	1.*Bifidobacteria, Akkermansia, Clostridia* ↑	TαMCA, TβMCA, TCA ↓ DCA ↑	(Han et al., 2020)
Plant herbs	Radix Scutellariae	2.5 g/kg/day by gavage for 28 days	HFD-diet SD Rat	liver index ↓ hepatic steatosis, inflammation and ballooning of hepatocytes ↓ serum TC ↓, TG ↓,LDL-C ↓ serum insulin ↓, HOMA-IR ↓	1.*Lactobacillus* ↓	1.DCA, LCA, GDCA, GLCA, GUDCA, TLCA ↑	(Zhao et al., 2021)
	Ganoderma lucidum	2.25 and 4.5 mg/mouse/day in diet for 43 days	HCD-diet C57BL/6J male mice	body weight ↓, liver weight ↓ hepatic steatosis, inflammation and ballooning of hepatocytes ↓ hepatic TC ↓, TG ↓ serum TC ↓, TG ↓, LDL-C↓ serum ALT ↓, AST↓	1.*Lactobacillus* ↑	1.Fecal BAs ↑	(Meneses et al., 2016)
		2.25 and 4.5 mg/mouse/day in diet for 43 days	HCD-diet C57BL/6J male mice RAW 264.7 cells treated under HC concentration	lipid droplet ↓ serum TC ↓, TG ↓, LDL-C↓	1.*Lactobacillaceae, Lactobacillus* ↑	1.BAs synthesis ↑	(Romero-Cordoba et al., 2020)
	Grifola frondosa	150 mg/kg/day by gavage for 8 weeks	HFD-diet SD Rat	body weight ↓ hepatic steatosis, inflammation and ballooning of hepatocytes ↓ hepatic TC ↓, TG ↓, LDL-C ↓, FFA ↓, HDL-C ↑ hepatic AST ↓ serum TC ↓, TG ↓, LDL-C ↓, HDL-C ↑ serum ALT ↓, AST↓	1.*Butyricimonas genus* ↑	*1.*Fecal BAs ↑	(Pan et al., 2018)

**Table 2:** Active ingredients in natural plants regulate the interplay between GM and BAs in the treatment of NAFLD.

Active ingredients in Plants		Dosage and administration	Model	Main Effects on NAFLD	Main Effects on Gut Microbiota	Main Effects on Bile Acids	Reference
Polysaccharide	Guar Gum and Pectin	24%, 70% Pectin in diet for 3 weeks	HFD-diet Wistar Rat	liver weight ↓ hepatic TC ↓, TG ↓ serum TC ↓, TG↓	an unclassified family in RF32 ↓ Oscillospira, Ruminococcaceae ↑	DCA, HDCA ↓ CA, CDCA, UDCA,α-, β-,ω-MCA ↑	(Ghaffarzadegan et al., 2016)
	Grifola frondosa polysaccharides	400 mg/kg/day by gavage for 8 weeks	HFD-diet Wistar Rat	body weight ↓ hepatic TG ↓, TC ↓, FFA ↓, AST ↓, ALT ↓ hepatic steatosis, inflammation and ballooning of hepatocytes↓ serum TC ↓, TG ↓, LDL-C ↓, FFA↓ serum ALT ↓, AST↓	Helicobater, Intestinimonas, Barnesiella, Defluviitalea, Ruminococcus, Flavonifractor, Paraprevotella ↑ Clostridium-XVIII, Butyricicoccus, Turicibacter ↓	1. fecal BAs ↑	(Li et al., 2019)
	Microalgae Chlorella pyrenoidosa	150, 300 mg/kg/day by gavage for 8 weeks	HFD-diet Wistar Rat	hepatic TG ↓, TC↓ hepatic steatosis, inflammation and ballooning of hepatocytes ↓ serum TC ↓, TG ↓, LDL-C ↓, HDL-C ↑	1.*Lactobacilli* ↑	1.fecal BAs ↑	(Wan et al., 2020)
Oligosaccharides	Citrus Pectin Oligosaccharides	0.15, 0.45, 0.9 g/kg/day by gavage for 8 weeks	HF-diet C57BL/6J male mice	hepatic TG ↓, TC↓ serum TC ↓, TG ↓, LDL-C↓	1.*Bifidobacterium*, *Lactobacillus*, *Bacteroides* ↑	1.fecal bile acid ↑	(Hu et al., 2019)
Polyphenols	Theabrownin	225 mg/kg/day by gavage for 30 days	HFD-diet C57BL/6J male mice	body weight ↓ hepatic TG ↓, TC ↓ serum TC ↓, TG	1.*Lactobacillus, Bacillus, Streptococcus, Lactococcus* ↓	TCDCA,CDCA↑ CA↓	(Huang et al., 2019)
	EGCG	0.632 ± 0.02 mg/mouse/day in diet for 8 weeks	HFD-diet C57BL/6J male mice	body weight ↓, liver weight ↓ hepatic TG ↓ hepatic steatosis, inflammation and ballooning of hepatocytes ↓	1.*Akkermansia, Parabacteroides* ↓	CA↓ TCA ↑	(Naito et al., 2020)
	Dicaffeoylquinic Acids	3.3, 10 mg/mouse/day by gavage for 8 weeks	HFD-diet C57BL/6J male mice	body weight, liver weight ↓ hepatic TG ↓ serum TC ↓, TG ↓, LDL-C ↓, HDL-C ↑	1.*Bifidobacterium, Akkermansia* ↑	induced functional differences of microbial communities consisted primary bile acid biosynthesis and secondary bile acid biosynthesis	(Xie et al., 2019)
	Pure total flavonoids from citrus	50 mg/kg/day by gavage for 12 weeks	HFD-diet C57BL/6J male mice	hepatic steatosis, inflammation and ballooning of hepatocytes ↓ serum TC ↓, TG↓ serum ALT ↓, AST↓	1.*Bacteroidaceae, Christensenellaceae* ↑	TDCA, DCA, TCA, CA ↓ the ratio of secondary to primary bile acids ↑	(He et al., 2021)
Polyketides	Monascus yellow, red and orange pigments	20 mg/kg/day by gavage for 8 weeks	HFD-diet Wister Rat	hepatic steatosis, inflammation and ballooning of hepatocytes ↓ hepatic TC ↓, TG ↓, FFA ↓ hepatic ALT ↓, AST ↓ serum TC ↓, TG ↓, LDL-C ↓, HDL-C ↑ serum ALT ↓, AST ↓	1. *Oscillibacter* sp.*, Ruminococcus albus, Clostridium sp* ↑	1.fecal BAs ↑	(Zhou et al., 2019b)
Alkaloids	Rhizoma Coptidis alkaloids	140 mg/kg/day by gavage for 35 days	HFHC-diet C57BL/6J male mice	body weight ↓ hepatic TG ↓ hepatic steatosis, inflammation and ballooning of hepatocytes ↓	Escherichia coli, Desulfovibrio C21_c20, Parabacteroides distasonis ↓ Sporobacter termitidis, Alcaligenes faecalis, Akkermansia muciniphila ↑	1.serum total BAs↓	(He et al., 2016)
Triterpenoids	2α-OH-Protopanoxadiol	200 mg/kg/day by gavage for 32 days	HFD-diet C57BL/6J male mice	body weight ↓, liver weight ↓ hepatic TG ↓ hepatic steatosis, inflammation and ballooning of hepatocytes ↓ serum TG ↓, TC ↓ Plasma glucose ↓, insulin ↓	1.*Brucella, Desulfovibrio, Bacteroidetes*↓	1.intestinal Tα/βMCA↑	(Xie et al., 2020)

### 3.1 Plants

#### 3.1.1 Food Plants

##### 3.1.1.1 Tea

Tea, processed from the leaves of *Camellia sinensis*, is one of the most widely consumed beverages worldwide. This drink is characterized by high levels of an antioxidant that has been shown to prevent different types of metabolic syndromes, cardiovascular diseases, obesity, and type 2 diabetes (Bag et al., 2022). Huang et al. indicated that consumption of Pu-erh tea significantly increased the content of fecal conjugated BAs by decreasing the BSH activity, which was linked to a reduction in the levels of *Lactobacillus*, *Bacillus*, *Streptococcus*, and *Lactococcus*, resulting in the inhibition of intestinal FXR-FGF15 activation. The excretion of BAs was enhanced in the feces as the expression of CYP27A1 and CYP7B1 increased in the liver, thereby reducing hepatic lipid deposition in HFD-fed mice (Huang et al., 2019).

Huangjinya black tea is a natural photosensitive tea containing abundant free amino acids. This drink has been proven to ameliorate hepatic steatosis by reducing hepatic triglyceride (TG), free fatty acid (FFA), and lipid accumulation in HFD-fed mice. Furthermore, Huangjinya black tea consumption could also significantly reverse GM disruption and increase the ratio of primary/secondary BAs (Xu et al., 2020).

##### 3.1.1.2 Whole Grain

Whole grains contain the endosperm, germ, and bran of the original seeds, in contrast to refined grains, in which the germ and bran are removed during the milling process (Aune et al., 2016). The whole grain, rich in dietary fiber, is considered to have a beneficial effect on the risk reduction of NAFLD (Berna and Romero-Gomez, 2020). Sun et al. confirmed that oats and tartary buckwheat-based food (OF) reduced the hepatic total cholesterol (TC) and TG levels in HFD-fed hamsters. Furthermore, it can change the overall structure of the gut microbiota. Specifically, the relative abundance of *Erysipelotrichaceae*, *Ruminococcaceae*, and *Lachnospiraceae* decreased, whereas that of *Eubacteriaceae* increased. Meanwhile, the excretion of fecal BAs increased and the concentration of SCFAs (acetic acid, propionic acid, butyric acid, and total short-chain fatty acids) increased significantly after OF intervention (Sun et al., 2019b).

Another study revealed that wheat gluten consumption resulted in higher fecal BA excretion, thereby reducing the hepatic TC and TG levels in HCD-diet hamsters. Moreover, the enhanced fecal BAs may be related to an increase in hepatic CYP7A1 levels. Furthermore, it has been shown that the relative abundance of *Firmicutes* and *Erysipelas* decreased, whereas that of *Bacteroides, Bacteroides*, and *Rumenococcus* increased (Liang et al., 2019).

##### 3.1.1.3 Legumes

Legumes are defined as the pods or fruits of plants belonging to the botanical families *Leguminosae* or *Favaceae*, which includes soybeans, peanuts, green/dry beans, and lentils (McCrory et al., 2010). These seeds are a good source of protein, fiber, B vitamins, minerals, and polyphenols, and are considered as low-glycemic-index foods (Rebello et al., 2014). Populations with high legume consumption (peas, beans, lentils) have a low risk of cancer and chronic degenerative diseases (Campos-Vega et al., 2013).

Micioni et al. found that lentils significantly reduced serum TC and LDL levels in HCD-fed rats. Furthermore, the contents of fecal BAs were enhanced, resulting in an increased excretion of BA in feces. In addition, the abundance of *Bifidobacteria* and the level of butyric acid markedly increased after intervention with lentils (Micioni Di Bonaventura et al., 2017). Inagaki et al. demonstrated the effect of pea pods on decreasing serum TC and TG levels in HS-fed SD rats. Meanwhile, the upregulation of fecal BAs and the abundance of bifidobacteria were observed significantly (Inagaki et al., 2016).

##### 3.1.1.4 Plant Oil

Plant oil, rich in dietary omega-3 long-chain polyunsaturated fatty acids (LCPUFA), comprises important biomolecules that have been shown to regulate hepatic lipid metabolism (Manson et al., 2019; Maattanen et al., 2020). For example, wild melon seed oil (CO) supplementation reduced the body weight, liver weight, and hepatic TC and enhanced the excretion of fecal BAs, upregulating the gene expression of hepatic CYP7A1 in HCD-fed hamsters. Meanwhile, CO intervention favorably altered the relative abundances of *Eubacteriaceae*, *Clostridiales_vadinBB*60_group, *Ruminococcaceae*, *Streptococcaceae*, and *Desulfovibrionaceae* at the family level (Hao et al., 2020).

In another study, oral consumption of sacha inchi oil alleviated hepatic fat accumulation in HFD-fed rats. Furthermore, the imbalance of the GM was reversed, embodied by decreasing abundances of *Enterobacteriaceae* and *Escherichia* and increasing ratios of *Roseburia*, *Turicibacter*, and *Butyrivibrio*. Meanwhile, the content of CA, GCA, TCDCA, and TCA significantly decreased after sacha inchi intervention (Li et al., 2020).

##### 3.1.1.5 Grapes

Grapes are one of the most highly consumed fruits worldwide. In addition to being a rich source of vitamins and fiber, the skin and seeds of grapes contain abundant polyphenols, specifically proanthocyanidins. The proanthocyanidin-rich grape seed extract is beneficial against many diseases, including inflammation, cardiovascular disease, hypertension, diabetes, cancer, peptic ulcers, and microbial infections.

Han et al. indicated that grape extract (GE) decreased liver weight, accompanied by a decline in adipocyte size and hepatic fatty deposits in HFD-fed mice. Additionally, GE restores dysbiosis of the GM by augmenting the observed species, enhancing the *Firmicutes*-to-*Bacteroidetes* ratio, and increasing the abundance of the *Bifidobacterium*, *Akkermansia*, and *Clostridia* genera. The restoration of *Akkermansia*, *Clostridium*, and *Bifidobacterium* was negatively correlated with the concentrations of TαMCA, TβMCA, and TCA, but positively correlated with DCA (Han et al., 2020).

#### 3.1.2 Plant Herbs

##### 3.1.2.1 Scutellaria baicalensis

*Scutellaria baicalensis* Georgi. (*Lamiaceae*) is a plant of the genus *Lamiaceae*, whose root is commonly used as a medicine. This medicinal plant is widely distributed in China, Russia, Mongolia, North Korea, and Japan. To date, over 40 compounds have been isolated and identified from *Scutellaria baicalensis*, including flavonoids, terpenoids, volatile oils, and polysaccharides. The compounds and extracts exhibit a wide range of pharmacological activities, including effects on the immune system, liver protection, antioxidant effects, and other pharmacological effects (Zhao et al., 2019).

Zhao et al. found that *Radix scutellariae* water extract (WESB) improved hepatic steatosis, inflammation, and ballooning of hepatocytes in HFD-fed rats. Of note, the abundance of *Lactobacillus*, which exhibits significant negative correlations with certain fecal-conjugated BAs (TCDCA, GUDCA, and TUDCA), was significantly decreased in the feces of HFD-fed rats after treatment with WESB. Moreover, this effect might be related to the activation of hepatic CYP7A1 and the inhibition of FXR expression in the intestine rather than in the liver (Zhao et al., 2021).

##### 3.1.2.2 Ganoderma lucidum (Gl)

Gl, commonly referred to as “Lingzhi” in Chins, is one of the best-known medicinal mushrooms, and has been used in herbal medicines worldwide for more than two thousand years. In recent decades, Gl*-*related biological and pharmacological research has focused on the bioactive compounds extracted from its fruiting bodies, including polysaccharides, triterpenoids, sterols, proteins, and peptides, which comprise constituents with numerous biological activities such as antioxidant, anti-inflammatory, immunomodulatory, hypoglycemic, and hypolipidemic activities (Lu et al., 2020).

Several studies have shown that the consumption of low and high doses of Gl extracts almost entirely prevented the accumulation of lipids in the liver of high-cholesterol diet-fed mice. Analyses of hepatic cholesterol and triglyceride levels confirmed this histological analysis. These effects were associated with a significant increase in the excretion of fecal bile acids and an increase in *CYP7A1* gene expression in the liver. Furthermore, the relative abundance of *Lactobacillus* significantly increased after intervention with Gl (Meneses et al., 2016).

Sandra et al. showed that Mexican Gl extracts prevent hepatic fatty acid synthesis and accumulation through the downregulation of genes involved in lipogenesis. Furthermore, the Gl extract activated one of the major BA synthesis pathways *via* CYP7B1-mediated hydroxylation of cholesterol. In addition to gene modulation, the administration of Gl extracts also modulates the composition of the gut microbiota, triggering an increase in abundance of the *Lactobacillaceae* family and the genus *Lactobacillus* (Romero-Cordoba et al., 2020).

##### 3.1.2.3 Grifola frondosa (GF)

*Grifola frondosa* (Dicks.) Gray is a widely consumed edible and medicinal fungus. Ancient books record that it can boost qi and fortify the spleen, moisten the lungs, and protect the liver. Over the past three decades, GF polysaccharides have been shown to possess various promising bioactivities, including antitumor, immunomodulation, anti-oxidation, and anti-hyperglycemia, and can also effectively act on the skin and hematopoietic stem cells (He et al., 2017).

Pan et al. found that the levels of serum ALT/AST and hepatic lipid accumulation were significantly decreased in GF-treated HFD-fed rats. Furthermore, GF consumption significantly enhanced the excretion of BAs in the cecum. In addition, a higher abundance of *Butyricimonas* was observed in the GF group (Pan et al., 2018).

### 3.2 Active Ingredients

Previously, we reviewed the efficacies and modes of action of plants regulating the interplay between GM and BAs in NAFLD. It is important to note that many of the active ingredients of these plants can exert the same medicinal effects as the source plant itself. Therefore, from the perspective of the chemical structure, we divided these active ingredients into polysaccharides, oligosaccharides, polyphenols, ketones, and other active ingredients.

#### 3.2.1 Polysaccharides

Polysaccharides, which are natural macromolecules composed of monosaccharides, are among the most important members of the biopolymer family. To date, more than 300 natural polysaccharide compounds have been identified and are ubiquitously present in plants, animals, and microorganisms, where they engage in a variety of physiological functions. Several pharmacological and clinical studies have shown that plant polysaccharides have multiple functions, such as immune regulation, anti-inflammatory, antiviral, and hypoglycemic (Kouakou et al., 2013; Chen and Huang, 2018).

##### 3.2.1.1 Guar Gum and Pectin

Guar gum, a naturally occurring polymer, is a galactomannan obtained from the ground endosperm of *Cyamopsis tetragonolobus* or *Cyamopsis psoraloides*. Pectin is a heteropolysaccharide abundant in the cell wall of plants and is obtained mainly from fruits (citrus and apple). In HFD-fed rats, guar gum and pectin significantly decreased hepatic TG and TC levels. Furthermore, guar gum increased the cecal amounts of CA, CDCA, and UDCA, as well as α-, β-, and ω-MCA to a greater extent, whereas that of DCA and HDCA were reduced. In contrast, differences in BA composition between pectin groups were less obvious, but the cecal levels of α- and ω-MCA were higher in rats. Meanwhile, these two fibers decreased the cecal abundance of *Oscillospira* and an unclassified genus in *Ruminococcaceae*, while increasing that of an unclassified family in RF32 (Ghaffarzadegan et al., 2016).

##### 3.2.1.2 G. frondosa Polysaccharides

*G. frondosa* polysaccharides (GFP), derived from the plant *Grifola frondosa*, exerts various biological activities, such as antitumor, hypoglycemic, antioxidant, and immune regulatory activities. Li et al. found that GFP markedly alleviated hepatic lipid accumulation and steatosis in HFD-fed rats. In addition, the excretion of fecal BAs was also promoted by significantly increasing the mRNA expression of *CYP7A1* and *BSEP* after oral administration of GFP. Meanwhile, GFP supplementation significantly increased the proportion of *Helicobater*, *Intestinimonas*, *Barnesiella*, *Defluviitalea*, *Ruminococcus*, *Flavonifractor*, and *Paraprevotella*, but decreased the relative abundances of *Clostridium-XVIII*, *Butyricicoccus*, and *Turicibacter* (Li et al., 2019).

##### 3.2.1.3 Polysaccharides From Chlorella pyrenoidosa (CPP)

The microalgae *Chlorella pyrenoidosa* is considered a valuable and nutritious microalga, which contains a variety of polysaccharides, carotene, chlorophylls, and polyunsaturated fatty acids in abundance. The pharmacological effects of *Chlorella* phytochemicals include anti-inflammatory, antitumor, and antioxidant activities. Moreover, CPP is regarded as an effective hypolipidemic agent. Wan et al. demonstrated that CPP could effectively inhibit hepatic lipid accumulation in HFD-fed rats. In addition, CPP accelerates the metabolism of total cecal BAs, while increasing the relative abundance of lactobacilli in the intestines (Wan et al., 2020).

#### 3.2.2 Oligosaccharides

Oligosaccharides are saccharide polymers containing a small number of monosaccharides, which are obtained by direct extraction from natural sources, hydrolysis of polysaccharides, or enzymatic and chemical synthesis from disaccharides; they play an important role in preventing obesity and other metabolic diseases (McCranie and Bachmann, 2014).

Hu et al. showed that administration of citrus pectin oligosaccharides (POS) markedly reduced liver weight and hepatic TC in HF-fed mice. In addition, the relative abundances of specific bacterial groups in the feces and the concentrations of their metabolites were higher after POS intervention. Furthermore, we observed significant correlations among *Bifidobacterium*, *Lactobacillus*, and *Bacteroides* and fecal BAs, as well as hepatic CYP7A1 reductase (Hu et al., 2019).

#### 3.2.3 Polyphenols

Polyphenols are a large and heterogeneous group of phytochemicals, including multiple sub-classes such as flavonoids, stilbenes, phenolic acids, and lignans (Manach et al., 2005), which have shown promise in the management of many diseases due to their antioxidant, anti-inflammatory, anti-fibrotic, and metabolic regulation functions. Several hundred different polyphenols are found in plant-based foods, including tea, coffee, legumes, cereals, plant-derived beverages, and chocolate (Khan et al., 2008).

Huang et al. indicated that HFD-fed mice receiving the polyphenol theabrownin exhibited significant decreases in hepatic TG and TC levels. Additionally, the abundance of BSH-producing *Lactobacillus*, *Bacillus*, *Streptococcus*, and *Lactococcus* genera decreased, as did the BSH activity in mice treated with theabrownin. Conjugated BAs inhibit intestinal FGF15/FGF19-FGFR4 signaling, coupled with increased activation of hepatic FXR-SHP signaling in the liver, thereby increasing CYP7B1 levels to promote BA synthesis (Huang et al., 2019).

##### 3.2.3.1 Phenolic Acids

Xie et al. revealed that Kudingcha dicaffeoylquinic acid (diCQA), made from Kudingcha, decreased the hepatic and adipose tissue masses in HFD-fed mice. It can also induce functional differences in microbial communities consisting of several metabolic pathways, including primary bile acid biosynthesis and secondary bile acid biosynthesis. Furthermore, the relative abundances of *Bifidobacterium* and *Akkermansia* were shown to increase after treatment with diCQAs (Xie et al., 2019).

##### 3.2.3.2 Flavonoids

Naito et al. confirmed that EGCG significantly inhibited the HFD-induced increase in histological fatty deposits and TG accumulation in the liver, and improved intestinal dysbiosis. Meanwhile, inhibition of 7a-dehydroxylation, associated with abundance of the *Akkermansia* and *Parabacteroides* genera, was shown to decrease the conversion of CA to TCA in the intestine (Naito et al., 2020).

He et al. found that after pure citrus total flavonoid (PTFC) intervention, the degree of fatty changes, infiltration of inflammatory cells, and ballooning of hepatocytes in the liver of HFD-fed mice were significantly reduced. Furthermore, while the relative abundances of *Bacteroides* and *Artemisiaceae* increased, the content of toxic BAs (TDCA, DCA, TCA, CA) and the ratio of secondary BAs/primary Bas were enhanced. This revealed that PTFC could alleviate NAFLD by co-regulating GM and BA metabolism (He et al., 2021).

#### 3.2.4 Polyketides

Polyketides consist of a large group of natural biomolecules that are normally produced by bacteria, fungi, and plants. These molecules are clinically important because of their anti-cancer, anti-microbial, anti-oxidant, and anti-inflammatory properties (Xu et al., 2021). Monascus pigments (MPs) from red yeast rice significantly ameliorate lipid metabolism disorders by inhibiting hepatic lipid accumulation and steatosis in HFD-fed rats. Furthermore, the excretion of fecal BAs was also promoted by oral administration of MPs. Meanwhile, some beneficial GM (such as *Oscillibacter* sp., *Ruminococcus albus*, and *Clostridium* sp.) were found to be negatively correlated with serum and hepatic lipid indicators (Zhou et al., 2019b).

#### 3.2.5 Alkaloids

Alkaloids, as a class of natural ingredients derived from traditional Chinese medicines, have previously been shown to inhibit proliferation, metastasis, and angiogenesis; change cell morphology; promote apoptosis and autophagy; and trigger cell cycle arrest (Liu et al., 2019).

*Rhizoma coptidis* (RC) alkaloids reduced body weight gain, serum TG, and hepatic lipid accumulation in HFHC-fed mice. Furthermore, RC alkaloid feeding significantly increased the abundance of *Sporobacter termitidis*, *Alcaligenes faecalis*, and *Akkermansia muciniphila* in the gut of mice, whereas that of *Escherichia coli*, *Desulfovibrio C21_c20*, and *Parabacteroides distasonis* was suppressed. The synthesis of bile acids was increased by the upregulation of CYP7A1 (He et al., 2016).

#### 3.2.6 Triterpenoids

Triterpenoids (TTP) are widely distributed in higher plants and are of interest because of their structural diversity and broad range of bioactivities. TTP possesses antioxidant, metabolic-regulating, immunomodulatory, and anti-inflammatory activities, suggesting its application as an alternative therapy in some chronic diseases (Ren and Kinghorn, 2019).

2α-OH-protopanaxandiol (GP2), a metabolite of gypenosides *in vivo*, has been shown to protect mice from HFD-induced obesity and improve glucose tolerance. Moreover, GP2 treatment inhibited the activity of BSH and decreased the abundance of species of the genera *Brucella* and *Desulfovibrio* from *Proteobacteria* and the genera *norank_f_Bacteroidales_S24-7_group* from *Bacteroidetes*, thereby increasing the content of TβMCA in the intestine. TβMCA induced GLP-1 production and secretion by reducing the transcriptional activity of nuclear FXR, thereby ameliorating metabolic syndrome (Xie et al., 2020).

## 4 Discussion

In this review, we discussed and summarized the new findings regarding the effects of natural plants on the regulation of interplay between GM and BA to improve NAFLD. We systemically discussed the joint action between the GM and BA in the pathogenesis of NAFLD. These components show a strong interrelationship. The GM regulates BA synthesis, transport, and reabsorption *via* expression of metabolites and FXR, while also promoting the conversion of primary BAs to secondary BAs *via* enzyme activity. Conversely, BAs can directly inhibit the growth of intestinal flora by acting on bacterial membranes. In addition, it can indirectly affect the structure and growth of intestinal bacteria. In addition, we comprehensively reviewed the management of joint action between GM and BAs in NAFLD, in terms of the evidence regarding Natural Plants. These Natural Plants could act as reference for further drug research and discovery to treat NAFLD.

In recent years, a number of studies have shown the potential of food and herb plants as treatments for diet-induced NAFLD. We summarized that the main ingredients in plant foods, which worked effectively, might be attributed to dietary fiber, plant protein, and LCPUFA. Meanwhile, frequent changes in the GM mainly include alterations in the abundance of *Bacteroides*, *Bifidobacterium*, *Parabacter*, *Prevotella*, and *Clostridium* after intervention with plant foods. However, the changes in BAs modulated by the plant food profile did not show regularity. In addition, plant herbs, such as *Scutellaria baicalensis*, *Rhizoma coptidis*, *Ganoderma lucidum*, and *Grifola frondosa*, can also significantly improve the disturbance of GM and BA spectra caused in NAFLD, most of which are anti-NAFLD drugs commonly used in clinical settings. Furthermore, the abundance of *Escherichia coli*, *Bacteroides*, *Lactobacillus*, and *Monascus* changed frequently after drug treatment. Notably, most drugs improved NAFLD by acting on hepatic CYP7A1, indicating that the BA synthesis pathway accounts for a large proportion of treatment with plant herbs. From the perspective of pathogenesis, both foods and drugs improved glucose/lipid metabolism, insulin resistance, and inflammation in NAFLD. Interestingly, alterations in lipid metabolism were the most obvious, indicating the importance of this pathway.

In addition, active ingredients, including polysaccharides, oligosaccharides, polyphenols, alkaloids, and triterpenoids, can effectively regulate joint action in NAFLD. In the intestine of NAFLD hosts, the active substances mainly regulate the relative abundance of *Deferrobacterium*, *Akkermania*, *Parabacter*, *Bifidobacterium*, *Clostridium*, *Bacteroides*, and *Desulfovibrio*. Meanwhile, the synthesis pathway of hepatic BAs and modification of primary BAs were significantly modulated after intervention with active ingredients. Also, hepatic lipid metabolism plays a major role in the effect on active ingredients against NAFLD.

However, some limitations in this field remain. Although natural plants have been shown to regulate the interplay of GM and BAs in NAFLD, most studies have not fully clarified the regulatory effect on the interaction between GM and Bas, except for theanbrownin, suggesting that the potential mechanisms of many natural plants need to be further explored. In addition, it remains unclear whether some natural plants can be used as clinical drugs or dietary supplements. Meanwhile, heterogeneity between NAFLD patients should be considered in the clinical setting. Furthermore, the stability and safety of the natural plants need to be confirmed both *in vivo* and *in vitro*. Nevertheless, more in-depth research should be conducted to explore the potential mechanism of the interaction between GM and BAs to better reveal the therapeutic effect of natural plants on NAFLD.

## Author Contributions

QF conceived this study. QS was responsible for organizing the literature and drawing chemical formulas. QS, XX, and ZA were in charge of consulting the literature. QS wrote the manuscript and drew the figures. QF and YH supervised the study and gave final approval of the version to be published. All authors critically participated in the discussion and commented on the manuscript.

## Funding

This work was supported by the Key projects of national natural fund of China (No. 81830119 to YH), the National Natural Science Foundation of China (No. 82174186 to YH; No. 82174040 to QF), the Shanghai Key Laboratory of Traditional Chinese Clinical Medicine (No. 20DZ2272200), and the Innovative Projects of Shanghai University of Traditional Chinese Medicine (No. Y2021081 to QS).

## Conflict of Interest

The authors declare that the research was conducted in the absence of any commercial or financial relationships that could be construed as a potential conflict of interest.

## Publisher’s Note

All claims expressed in this article are solely those of the authors and do not necessarily represent those of their affiliated organizations, or those of the publisher, the editors and the reviewers. Any product that may be evaluated in this article, or claim that may be made by its manufacturer, is not guaranteed or endorsed by the publisher.

## Abbreviations

α-MCA, α-murocholic acid; ALT, alanine aminotransferase; adenosine; AST, serum aspartate aminotransferase; ASBT, apical sodium-dependent bile acid transporter; BAs, bile acids; BSH, bile salt hydrolase; β-MCA, β-murocholic acid; CA, Cholic acid; CDCA, Chenodeoxycholic acid; CYP7A1, cytochrome P450 family 27 subfamily A member 1; CYP7B1, cytochrome P450 family 7 subfamily B member 1; DCA, deoxycholic acid; FXR, farnesoid X receptor; FFA, free fatty acid; GM, gut microbiota; HDL-C, high-density lipoprotein cholesterol; HFD, high-fat diet; HFHC, high-fat high-calorie; IBABP, ileal bile acid binding protein; LCA, lithocholic acid; LDL-C, low-density lipoprotein cholesterol; NAFL, non-alcoholic fatty liver; NAFLD, non-alcoholic fatty liver disease; NAS, NAFLD activity score; NASH, non-alcoholic steatohepatitis; OCA, obeticholic acid; OST, organic solute transporter;SCFAs, short-chain fatty acids; SHP, small heterodimer partner; SREBP-1, sterol regulatory element binding protein-1; TβMCA, tauro-β-muricholic acid; TC, total cholesterol; TG, triglyceride; TMAO, trimethylamine oxide; UDCA, ursodeoxycholic.
